# Transcatheter arterial chemoembolization (TACE) combined with γ-knife compared to TACE or γ-knife alone for hepatocellular carcinoma

**DOI:** 10.1097/MD.0000000000010890

**Published:** 2018-06-01

**Authors:** Yeyu Cai, Qian Chang, Enhua Xiao, Quan-Liang Shang, Zhu Chen

**Affiliations:** Department of Radiology, The Second Xiangya Hospital of Central South University, Changsha, Hunan Province, China.

**Keywords:** γ-ray, conformal radiotherapy, primary hepatocellular carcinoma, transcatheter arterial chemoembolization

## Abstract

To compare the clinical efficacies and adverse reactions between transcatheter arterial chemoembolization (TACE), γ-ray 3-dimensional fractionated stereotactic conformal radiotherapy (FSCR), and TACE combined with FSCR for primary hepatocellular carcinoma.

The study was approved by the Institutional Review Board, and informed consent was waived due to the retrospective study design. About 121 patients met the inclusion criteria and were included in this study, from March 2008 to January 2010, in the Second Xiangya Hospital. Forty-six patients underwent TACE alone, 36 patients underwent γ-knife alone, and 39 were treated by γ-knife combined with TACE. Short-term effects, overall survival rates, adverse reactions, and survival times were compared between the 3 treatment groups.

Short-term effects were observed in 41.3% of the TACE group, 33.3% of the γ-knife group, and 64.1% of the TACE combined γ-knife group (*P* = .020). Overall survival rates at 6,12, 18, and 24 months were 50%, 34.8%, 28.3%, and 21.7% for the TACE group, 36.1%, 30.6%, 16.7%, and 11.1% for γ-knife group, and 84.6%, 71.8%, 61.5%, and 30.8% for TACE combined γ-knife group, respectively. The differences in the overall survival rates at 6, 12, and 18 months between the 3 groups were statistically significant (*P* = 0), but the overall survival rates at 24 months in the 3 groups were not significantly different (*P* = .117). The median survival time was 7 months for the TACE group, 3 months for the γ-knife group, and 20 months for the TACE combined γ-knife group (*P* = 0). There were statistically significant differences (*P* = .010) of leukopenia between the 3 groups, and no statistically significant differences of (*P* > .05) thrombocytopenia, anemia, nausea, vomiting, and liver function lesions.

TACE combined with γ-knife for primary hepatocellular carcinoma is superior to TACE or γ-knife alone in short-term and long-term effects. This procedure is a mild, safe, and effective treatment for primary hepatocellular carcinoma.

## 1 Introduction

Hepatocellular carcinoma (HCC) is one of the most common malignancies in China and is the sixth most frequent cause of cancer-related mortality worldwide.^[1–3]^ Only 20% to 30% of patients with HCC have the opportunity for curative procedures, including surgical resection and liver transplantation,^[4]^ and another 10% to 15% of patients are eligible for thermal ablation therapy.^[5,6]^ Male and female incidences of HCC were 14.7% and 4.9% per 100,000 individuals, after adjustment. Transcatheter arterial chemoembolization (TACE) has become the preferred method for patients who are not suitable for resection or ablation, in the absence of extrahepatic metastasis or early liver disease.^[7]^

The TACE is a combination of chemotherapeutic drugs and embolization agents that are administered into the hepatic artery. Embolization agents can embolize the distal branches of the tumor tissue, block blood supply, necrose or shrink the tumor tissue, reduce the rate of chemotherapy drug elimination, keep the normal liver tissue from experiencing serious damage by reducing the blood flow through the tumor cells, and increase the contact time between the drugs and the tumor. However, embolic agents do not completely destroy all tumor cells, especially the peripheral cells. The slow release of chemotherapy drugs plays a role in the continuous killing of tumor cells and significantly reduces systemic drug concentrations and adverse reactions. However, TACE cannot extend the survival rate of HCC upon tumor metastasis and the regrowth of residual tumor cells. After TACE, about 70% to 80% of patients die, and therefore, while the short-term efficacy of TACE for primary liver cancer is acceptable, the long-term efficacy is poor.^[8]^

Gamma knife (γ-knife) treatment is a special form of stereotactic radiation therapy that applies advanced computer technology for imaging, treatment planning, radiotherapy implementation, and verification to keep the radiation dose distribution consistent with the solid shape of the tumor tissue. Additionally, the γ-knife treatment greatly reduces the radiation dose to the surrounding normal tissue, while providing a much higher dose to the tumor tissue, in comparison with conventional radiotherapy. This enhances local tumor control rates and improves survival rates.^[9]^

The TACE for primary HCC has a high recent relief rate and was considered to be the first choice for non-surgical treatment, although the long-term efficacy is unsatisfactory. Stereotactic radiotherapy for tumor local control is good, and the incidence of treatment complications is acceptable. γ-Knife is a special form of stereotactic radiotherapy, and our research aims to combine TACE and γ-knife therapy to complement the advantages of each and explore the clinical value as a treatment for primary HCC.

## 2 Methods

### 2.1 Patients

In this retrospective study, 121 patients with HCC were enrolled, after meeting the inclusion and exclusion criteria, from March 2008 to January 2010. Forty-six cases were treated with TACE, 36 cases with γ-knife, and 39 cases with TACE combined with γ-knife. All subjects underwent TACE or γ-knife at the Second Xiangya Hospital and the Hunan Armed Police Corps Hospital.

### 2.2 Inclusion criteria

The inclusion criteria were as follows:

Adults who were 18 to 80 years of age, with primary HCC that was diagnosed in line with the domestic primary HCC clinical diagnosis and staging criteria or was confirmed by histopathology.

Patients with sufficient nutritional status, karnofsky performance status (KPS) ≥60, and life expectancies >3 months.

No serious complications (hypertension, coronary disease, or mental disease).

Liver function in Child-Pugh A or B, with normal renal and coagulation function.

No complete portal vein obstruction.

Tumor occupies <75% of the liver.

### 2.3 Exclusion criteria

The exclusion criteria were as follows:

Patients with severe hepatocellular jaundice, refractory ascites, liver failure, uncontrollable infections, or irreparable coagulation dysfunction.

Pregnant or in lactation.

General condition failure and life expectancy of <3 months.

The demographic and clinicopathologic characteristics of both groups are reported in Table 1, which shows that the 3 groups are not statistically different in terms of sex, age, tumor stage, tumor number, and liver function (*P* > .05). This study was approved by our institutional review board.

**Table 1:**
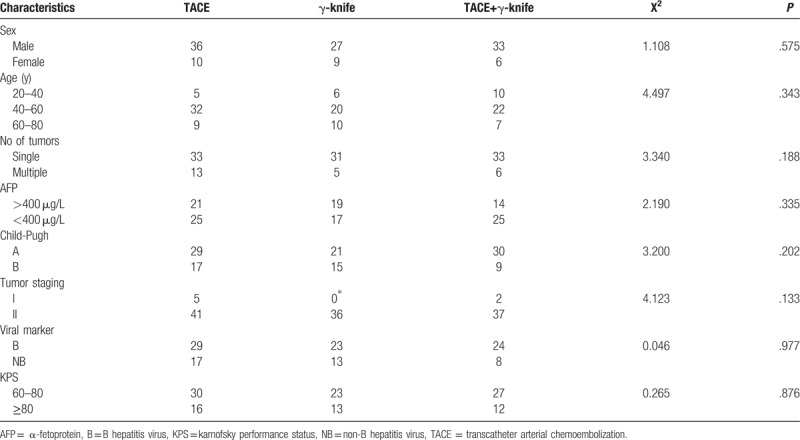
Demographic and clinicopathologic characteristics of patients with HCC, conforming to the Milan criteria.

### 2.4 Treatment protocols

#### 2.4.1 TACE procedure

All patients underwent percutaneous puncture of the femoral artery using the Seldinger method. A visceral angiography was carried out to assess the arterial blood supply to the tumor. A selective catheter was introduced into the arteria hepatica propria or the left and right hepatic arteries, according to tumor diameter and blood supply. Hepatic artery infusion chemotherapy was performed by specifically using nedaplatin/lobaplatin (80–100 mg), epirubicin (20–40 mg), 5-fluorouracil (1000–1500 mg), or mitomycin C (10–20 mg), according to tumor size and liver function. Meanwhile, the chemotherapy drugs were mixed with ultra-fluid lipiodol, compounding an emulsifier for embolization. For some cases, embolization was performed with absorbable gelatin sponge particles or polyvinyl alcohol with specific conditions, 4 to 8 weeks repeated, for a total of 1 to 3 times.

#### 2.4.2 γ-Knife procedure

The SGS-I stereotactic gamma ray system was used for treatment planning. A vacuum pillow with abdominal compression was used to fix the body, and at this time, resting expiratory levels were acquired using a contrast-enhanced computed tomography (CT) scan, with a slice thickness of 3 mm for each location. The CT scan ranged from 3 cm above the diaphragm to the inferior pole of the right kidney. The image and related data that were obtained were input into the treatment planning system. Two radiology oncologists reconstructed the 3-dimensional picture together and delineated the gross tumor volume (GTV). For determination of the planning target volume (PTV), individualized margins of 5 mm were applied to the exterior of the GTV, as a setup margin. The organs at risk were delineated by clinicians and physicists and included the normal liver tissue, pancreas, kidney, and medulla spinalis. We clinically adjusted the dose distribution, so that 50% to 70% of the isodose curve covered the PTV, and the normal tissue did not exceed tolerance amount. We evaluated and optimized the treatment plans using dose-volume histograms.

Radiation therapy was prescribed at a single dose of 250 to 400 cGy, every other day, 4 times a week, for 10 to 15 total treatments, with total tumor doses of 35 to 42 Gy. The shape of the field was designed by the beam eye view to ensure that the tumor target was within the field and to avoid the duodenum, pancreas, kidney, stomach, spinal cord, and other important organs as much as possible. During the course of the treatment, we regularly reexamined routine blood work and liver and kidney functions to ensure the best supportive care.

#### 2.4.3 TACE+γ-knife procedure

Patients underwent TACE 1 to 3 times, after which the γ-knife treatment was performed. After 2 weeks of rest, procedures were compared between with TACE and γ-knife groups.

### 2.5 Patient follow-up and statistics

Over the follow-up period of 3 to 32 months after treatment, the follow-up rate was 100%. The follow-up period was defined as a reexamination every 3 months for the year following treatment and every 6 months after the first. The follow-up included the improvements in clinical manifestations and signs, blood routines, α-fetoprotein (AFP) levels, liver and renal function, ultrasonic-B abdominal examinations, CT or magnetic resonance imaging scans, and improvement of quality of life.

For statistical analyses, we used SPSS 17.0 statistical software, and the Kaplan–Meier method was used to analyze the survival rates of the 3 treatment groups. To compare survival times, survival rates were analyzed using the log-rank test. The prognostic factor analysis was performed using the Cox's proportional hazards regression model, and *P* < .05 were considered statistically significant.

## 3 Results

### 3.1 Short-term efficacy

All patients were successfully treated with the 2 techniques. Three patients showed complete response (CR), 16 patients showed partial response (PR), 24 showed stable disease (SD), and 3 showed progressive disease (PD) in the TACE group. The overall response rate of the TACE group (CR+PR)/n, where n is the total number of patients, was 41.3% (19/46). In the γ-knife group, 3 patients showed CR, 9 showed PR, 15 showed SD, and 9 showed PD; the overall response rate was 33.3% (19/36).

However, in the TACE+γ-knife group, 6 patients showed CR, 19 showed PR, 7 showed SD, and 7 showed PD; the overall response rate was 64.1% (25/39). The differences between 3 groups were statistically significant (*P* < .05) (Table 2).

**Table 2:**
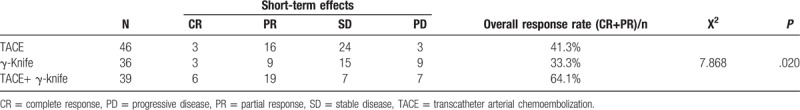
Comparison of the short-term effects between TACE, γ-knife, and TACE+ γ-knife groups.

### 3.2 Long-term effects

The 6-, 12-, 18-, and 24-month survival rates for the TACE group were 50% (23/46), 34.8% (16/46), 28.3% (13/46), and 21.7% (10/46), respectively. The 6-, 12-, 18-, and 24-month survival rates for the γ-knife group were 36.1% (13/36), 30.6% (11/36), 16.7% (6/36), and 11.1% (4/36), respectively. The 6-, 12-, 18-, and 24-month survival rates for the TACE combined with γ-knife group were 84.6% (33/39), 71.8% (28/39), 61.5% (24/39), and 30.8% (12/39), respectively. The differences between the 6-, 12-, and 18-month survival rates of the 3 groups were statistically significant (*P* < .05); however, the differences between the 24-month survival rates of the 3 groups were not statistically significant (*P* > .05) (Table 3).

**Table 3:**
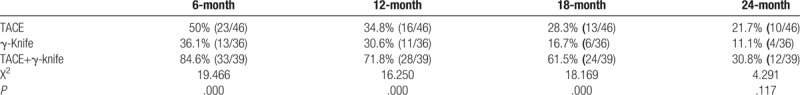
Comparison of the long-term effects between transcatheter arterial chemoembolization (TACE), γ-knife, and TACE+ γ-knife groups.

### 3.3 Complications

In all the 3 groups, the major complications were hypoleukocytosis, thrombocytopenia, anemia, nausea, vomiting, and liver function impairment. In the TACE group, the incidences of these major complications were 8.7% (4/46), 23.9% (11/46), 13.0% (6/46), 60.9% (28/46), and 65.2% (30/46), respectively. In the γ-knife group, the incidences were 22.2% (8/36), 25.0% (9/36), 16.7% (6/36), 66.7% (24/36), and 58.3% (21/36), respectively. In the TACE+γ-knife group, the incidences were 35.9% (14/39), 43.6% (17/39), 17.9% (7/39), 66.7% (26/39), and 66.7% (26/39), respectively. The differences between the incidences of hypoleukocytosis of the 3 groups were statistically significant (*P* < .05), but the differences between the incidences of thrombocytopenia, anemia, nausea, vomiting, and liver function impairment of the 3 groups were not statistically significant (*P* > .05) (Table 4).

**Table 4:**
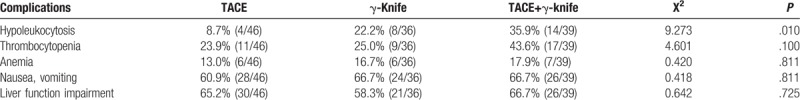
Comparison of complications between the transcatheter arterial chemoembolization (TACE), γ-knife, and TACE+γ-knife groups.

### 3.4 Survival time

**Figure 1:**
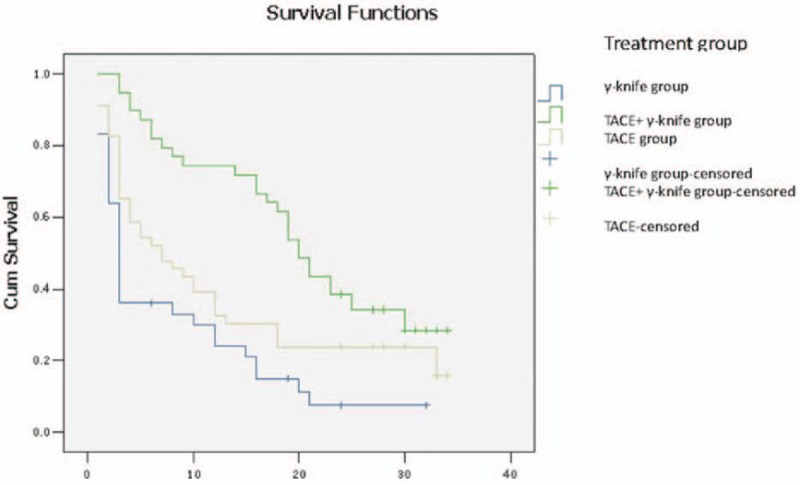
Survivorship curve of the 3 groups (months). Blue line: γ, knife treatment group cumulative survival rate. Green line: transcatheter arterial chemoembolization (TACE)+γ, knife group cumulative survival rate. Yellow line: TACE group cumulative survival rate. Blue cross: γ, knife-censored cumulative survival rate. Green cross: TACE+γ, knife-censored cumulative survival rate. Yellow cross: TACE-censored cumulative survival rate. The survival times of the 3 groups were statistically significantly different (X^2^ = 19.643, *P* = .000). The median survival time of the TACE, γ-knife, and TACE+γ-knife groups was 7, 3, and 20 months, respectively.

The Kaplan–Meier method was used to analyze the survival time of the 3 groups of patients. We found that the survival times of the 3 groups were statistically significantly different (X^2^ = 19.643, *P* = .000). The median survival time of the TACE, γ-knife, and TACE+γ-knife groups were 7, 3, and 20 months, respectively (Fig. 1).

### 3.5 Prognostic factors

By using the Cox proportional hazards regression model for a multivariate analysis of sex, age, tumor type, tumor number, tumor stage, HBsAg, AFP level, Child-Pugh, KPS score, and other basic characteristics of the TACE+γ-knife group, we found that that the tumor number, Child-Pugh, and tumor stage were the main factors that affected prognosis (*P* < .05). Age, sex, AFP, tumor type, and HBsAg had minimal effects on prognosis, which were not statistically significant (*P* > .05) (Table 5).

**Table 5:**
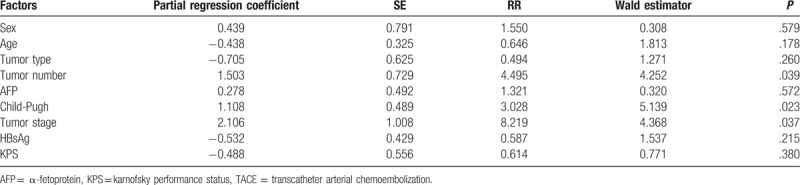
Cox model of multiple regression analyses of TACE+γ-knife (stepwise method, α=0.05).

## 4 Discussion

The early primary HCC symptoms are not obvious and lack characteristics. Patients visit the doctor mostly in the moderate and advanced stages, and at this time, have lost the optimal window for surgical resection. There are many non-operative treatments for HCC, but the best one is still controversial.

Liver tissue has its own unique physiologic regulatory action, and when one of liver blood supply system reduces, the other blood supply system compensatorily increases.^[10]^ Primary HCC is a hypervascular tumor that mostly receives nutrients from the hepatic arterial blood supply.

The mechanism of TACE for HCC involves the delivery of chemotherapy drugs to the tumor site through the feeding artery, which plays a role in killing tumor cells. Simultaneously, embolization agents are used to block the tumor-feeding artery, which results in endothelial cell injury of the feeding vessels and accelerates tumor tissue ischemic necrosis.^[11]^ Although most of the tumor lesions experience coagulation necrosis after embolization, complete necrosis is rare, and the surviving tumor cells^[12]^ are considered to be the source of recurrences after TACE.

From the treatment perspective, on one hand, tumor tissue will produce a certain resistance to chemotherapy drugs, and on the other hand, after embolization, part of the tumor tissue will recover blood supply.^[13]^ Therefore, while the short-term efficacy of TACE is good, this therapy has its limitations, and the long-term efficacy remains unsatisfactory.

The treatment of primary HCC is severely restricted due to the poor radiation tolerance of the liver (total liver irradiation tolerance dose <35 Gy). Since the surrounding normal tissue irradiation area is large, it is difficult to increase the dose to 35 Gy or more to the target, and this limits the local control rate of the tumor. In recent years, with the development of imaging technology and radiotherapy equipment, stereotactic conformal radiography (SBRT) has been widely used in clinical practice. A number of studies have confirmed that SBRT is a safe and effective treatment for HCC.^[14,15]^ The γ-knife is a specific form of stereotactic radiotherapy that uses the ^60^Co to deliver 18 Gy as the radioactive source and produces 30 beams of γ-rays that irradiate through a non-coplanar cone rotation over a fan-shaped of 360°. This highly concentrates the target dose into a maximum focal spot and reduces the surrounding dose, so that the normal tissue irradiation dose is reduced.^[16]^ The γ-knife is a filled, 3-dimensional conformal radiotherapy. The high radiation distribution dose is highly consistent with the 3-dimensional tumor target, and the tissues outside of the target area are only subject to scanning exposures during the radiotherapy process. Repeated radiation segmentation is also consistent with the biologic requirements of malignant tumors, and the treatment course is shorter, which reduces residual tumor cell proliferation and growth opportunities and improves the subsistence condition of patients.

Due to the unique biologic characteristics of liver cancer, any single treatment model, such as TACE or γ-knife alone, has limitations. Therefore, TACE combined with γ-knife treatment has become one of the most important non-surgical treatments for HCC.^[17]^ This study shows that the treatment efficacy of TACE combined with γ-knife is good. The total short-term effective rate was 64.1%, which was significantly better than the short-term efficacy of the TACE alone (41.3%) and γ-knife alone (33.3%) groups. The long-term 6-, 12-, 18-, and 24-month survival rates of the TACE+γ-knife group were 84.6%, 71.8%, 61.5%, and 30.8%, respectively, compared to 50%, 34.8%, 28.3%, and 21.7% of the TACE alone group and 36.1%, 30.6%, 16.7%, and 11.1% of the γ-knife alone group, respectively, which also showed certain clinical values for long-term efficacy.

Our sample size was small and there may be bias in patient sampling, data collection, etc. Therefore, our findings need to be further investigated in studies with larger sample sizes. Furthermore, our follow-up was limited, and this should be further increased in future studies.

In conclusion, TACE combined with γ-knife for primary HCC is a superior therapy to TACE or γ-knife alone in both short-term and long-term effects. This procedure provides a mild, safe, and effective treatment for patients with primary HCC.

## Author contributions

Yeyu Cai, Qian Chang, Enhua Xiao, designed research; Yeyu Cai, Qian Chang, Enhua Xiao, Quan-Liang Shang, Zhu Chen, performed research; Yeyu Cai, Qian Chang, Quan-Liang Shang, analyzed data; Yeyu Cai, Qian Chang, wrote the paper.

**Data curation:** yeyu cai, qian chang, QuanLiang Shang, Zhu Chen.

**Formal analysis:** yeyu cai, Zhu Chen.

**Writing – original draft:** yeyu cai, qian chang.

**Writing – review & editing:** yeyu cai.

**Conceptualization:** Enhua Xiao.

**Software:** qian chang.
